# Pterostilbene Attenuates Hexavalent Chromium-Induced Allergic Contact Dermatitis by Preventing Cell Apoptosis and Inhibiting IL-1β-Related NLRP3 Inflammasome Activation

**DOI:** 10.3390/jcm7120489

**Published:** 2018-11-27

**Authors:** Bour-Jr Wang, Hui-Wen Chiu, Yong-Lin Lee, Chia-Yi Li, Ying-Jan Wang, Yu-Hsuan Lee

**Affiliations:** 1 Department of Cosmetic Science and Institute of Cosmetic Science, Chia Nan University of Pharmacy and Science, Tainan 71710, Taiwan; pochih.wang@msa.hinet.net; 2 Department of Occupational and Environmental Medicine, National Cheng Kung University Hospital, Tainan 70403, Taiwan; 3 Division of Nephrology, Department of Internal Medicine, Shuang Ho Hospital, Taipei Medical University, New Taipei City 23561, Taiwan; leu3@tmu.edu.tw; 4 Graduate Institute of Clinical Medicine, College of Medicine, Taipei Medical University, Taipei 11031, Taiwan; 5 Department of Environmental and Occupational Health, College of Medicine, National Cheng Kung University, Tainan 70101, Taiwan; valley145@hotmail.com; 6 Honors in Neuroscience, Neuroscience and Mental Health Institute, Faculty of Science, University of Alberta, Edmonton, AB TG62R3, Canada; kronig1860@gmail.com; 7 Department of Medical Research, China Medical University Hospital, China Medical University, Taichung 40402, Taiwan; 8 Department of Food Safety/Hygiene and Risk Management, College of Medicine, National Cheng Kung University, Tainan 70101, Taiwan

**Keywords:** hexavalent chromium, pterostilbene, endoplasmic reticulum stress, inflammatory cytokines, NLRP3 inflammasome, allergic contact dermatitis

## Abstract

Hexavalent chromium (Cr(VI)) is widely used in many industries but can induce contact dermatitis especially in cement industries. Many cement workers suffer from Cr(VI)-induced allergic contact dermatitis (ACD), and prevention and therapeutic strategies are still lacking. Pterostilbene (PT) is a natural compound predominantly found in blueberries. Studies indicate the potential use of PT as an effective anti-oxidative and anti-inflammatory agent. Herein, we investigated the possible mechanisms involved and whether chromium-induced ACD could be effectively inhibited by treating PT. In our in vivo study, epidermal Cr(VI) administration causes cutaneous inflammation in mice ear skin, and the pro-inflammatory cytokines, TNF-α and IL-1β, were found in the epidermis, presenting the level of increase after Cr(VI) treatment. Meanwhile, the results of our in vitro experiment showed that apoptosis and endoplasmic reticulum (ER) stress were induced after treatment with different concentrations of Cr(VI) in HaCaT cells (human keratinocyte). Cr(VI) also induced TNF-α and IL-1β mRNA expressions, through the activation of the p38 mitogen-activated protein kinase (MAPK)/MAPK-activated protein kinase 2 (MK2) pathway. Notably, the severity of the skin reactions in the epicutaneous elicitation test significantly diminished when the mouse was treated with PT. Likewise, PT intervention also ameliorated the inflammation and apoptosis of HaCaT cells in vitro. Furthermore, our current findings demonstrated that the NLRP3 inflammasome could be involved in the Cr(VI)-mediated inflammation and apoptosis of ACD. Thus, interrupting this mechanism with proper nontoxic agents, such as PT, could be a new option to improve occupational chromium toxicity and hypersensitivity.

## 1. Introduction

Allergic contact dermatitis (ACD) is an important occupational disease caused mainly by topical exposure to hexavalent chromium (Cr(VI)), an impurity of cement manufacture. In Europe, the prevalence of ACD is approximately 4–5% in cement workers [1]. In some Asian countries the prevalence is even higher, such as 40% in Singapore and 13% in Taiwan [2,3]. Due to its high prevalence, ACD exerts an important occupational health impact on those workers. The pathologic process of ACD has been well defined as a cell-mediated type IV hypersensitivity reaction in which many cell types are involved, including keratinocytes, dendritic cells, T-lymphocytes and leucocytes [4]. Reactive oxygen species (ROS) activation was proposed as the initial event, as it leads to the activation of transcriptional factors and signaling pathways, including NF-κB and p38 mitogen-activated protein kinase (MAPK) pathway, which leads to the release of pro-inflammatory cytokines such as TNF-α and IL-1β [5,6,7]. Interestingly, the p38-downstream kinase MAPK-activated protein kinase 2 (MK2) has recently been explored in inflammatory disorders of the skin, such as psoriasis and has become a therapeutic strategy for anti-inflammatory disease. Disruption of MK2 signaling leads to a significant reduction in the level of several pro-inflammatory cytokine production [8]. ROS are also involved in the activation of the NLRP3 inflammasome, which is required to direct the proteolytic maturation of inflammatory cytokines, such as IL-1β and IL-18. Recent studies have indicated that the NLRP3 inflammasome is often wrongly activated by environmental irritants, thus resulting in cutaneous inflammatory diseases [9]. Moreover, ROS can facilitate the process of ACD by changing the extracellular microenvironment [10]. When skin is exposed to Cr(VI), it can penetrate and bind to keratinocytes and immune cells. The intracellular reduction of Cr(VI) is associated with the production of ROS, leading to dermal toxicity, cytokine excretion and skin hypersensitivity [1].

In our previous studies, we demonstrated that Cr(VI) can increase intracellular ROS generation and activate the NF-κB, Akt and MAPK pathways, leading to increased production of TNF-α and IL-1α in keratinocyte in vitro and in a guinea pig in vivo [11]. In addition, Cr(VI) can also induce both apoptotic cell death and endoplasmic reticulum (ER) stress during ROS-triggered cytotoxicity [12,13]. ER is an organelle responsible for protein folding and assembly. Most newly synthesized proteins translocate to the lumen of the ER and fold into the correct three-dimensional structures. However, excessive ROS production disrupts the ER function and results in the accumulation of unfolded or misfolded proteins in the ER lumen, also known as ER stress [14]. ER stress has been implicated in many diseases, including subclinical cutaneous inflammation [15]. Substantial ER stress can interfere with Ca^2+^ homeostasis and lead to the inflammatory response and cell death in a variety of cell types [16]. Apoptotic cell death of keratinocytes has also been proven to play an important role in skin inflammatory diseases [17]. In this regard, there could be a direct link among ER stress, cell death, antigen processing, and the generation of inflammatory and immune responses [14,18,19]. However, to the best of our knowledge, no study has examined the interactive effects of apoptosis, ER stress and NLRP3 inflammasome during the pathogenesis of Cr(VI)-induced ACD.

Pterostilbene is a naturally occurring chemical classified as a stilbene, predominantly found in blueberries, with multiple benefits in the treatment and prevention of human disease based on its anti-oxidative, anti-inflammatory, and anti-carcinogenic properties [20]. The anti-oxidative effects of pterostilbene could be derived from its unique structure that may scavenge extracellular ROS [21]. These effects provide the opportunity for pterostilbene to diminish free radical-triggered tissue damage during chronic inflammation. In addition to its ROS scavenging effects, pterostilbene can also regulate several inflammation-related molecular targets, including inducible nitric oxide synthase (iNOS), cyclooxygenases (COX), NF-κB, TNF-α, interleukins (ILs) and many more [22]. This anti-oxidative and anti-inflammatory activity is believed to stand behind of its positive health effects against skin disease [22].

Despite preclinical evidence showing that pterostilbene may have diverse pharmacological benefits for the prevention and treatment for a vast range of human diseases, including skin disorders [23], little is known about how or even whether pterostilbene can effectively prohibit Cr(VI)-induced ACD and its possible underlying mechanisms. Therefore, the purposes of this study were to validate the role of pterostilbene in Cr(VI) hypersensitivity using a C57BL/6 mice animal model, to examine the biomarkers of TNF-α and the NLRP3 inflammasome (IL-1β) in keratinocytes in vitro and in mouse skin in vivo treated with Cr(VI) alone or in combination with pterostilbene and to explore the involved signaling regulators, such as apoptosis, ER stress and p38/MK2 kinase. Our results clearly demonstrated that pterostilbene attenuated Cr(VI)-induced skin inflammation in mice through the inhibition of TNF-α and the NLRP3-inflammasome (IL-1β) and consequent cell death by apoptosis. Thus, pterostilbene could be a potential chemopreventive agent for the prevention of Cr(VI)-induced cytotoxicity and ACD.

## 2. Experimental Section

### 2.1. Reagents

Dulbecco’s modified Eagle’s medium (DMEM), penicillin, and streptomycin were purchased from Gibco BRL (Grand Island, NY, USA). Dimethyl sulphoxide (DMSO) and ethylenediaminetetraacetic acid (EDTA) were purchased from Sigma Chemical (Poole, Dorset, UK). Potassium dichromate (K_2_Cr_2_O_7_, Cr(VI)) and *N*-acetylcysteine (NAC) were obtained from Merck Chemical Co. (Darmstadt, Germany). MTT (3-(4,5-dimethylthiazol-2-yl)-2,5-diphenyltetrazolium bromide), Dimethyl sulphoxide (DMSO), EDTA, β-Cyclodextrin and Freunds complete adjuvant (FCA) containing Mycobacterium butyricum, 6-hydroxy-2,5,7,8-tetramethylchroman-2-carboxylic acid (Trolox), 2,2¢-azobis (2-amidinopropane) dihydrochloride (AAPH), b-phycoerythrin (b-PE), horseradish peroxidase (HRP) and butylated hydroxyl toluene (BHT) were obtained from Sigma–Aldrich (St. Louis, MO, USA). Pterostilbene (PT) was provided by Dr. Chi-Tang Ho (Department of Food Science, Rutgers University, New Brunswick, NJ, USA). PT was synthesized according to the method reported by Pettit et al. [24] and the purity of pterostilbene was determined by high performance liquid chromatography (HPLC) as higher than 96% (Figure S1).

### 2.2. Mice

All experiments on mice were performed according to the guidelines of our institute (Guide for Care and Use of Laboratory Animals, National Cheng Kung University Medical College) and were approved by the Institutional Animal Care and Use Committee of National Cheng Kung University, Taiwan (Approval No.: 103159). Specific pathogen-free female C57BL/6 mice were obtained from the National Cheng Kung University Medical College. Animals were 8–12 weeks of age at the onset of experiments. They were acclimatized for 1 week prior to the start of the experiments and had free access to drinking water (tap water) and standard rodent laboratory chow (no. 5001; Laboratory Rodent Diet, USA). The animals were housed five per cage at 24 ± 2 °C with 50% ± 10% relative humidity and subjected to a 12-h light/dark cycle.

### 2.3. Administration of PT and Sensitization of Mice

PT was dissolved in 5% β-Cyclodextrin, which can improve the solubility and bioavailability of PT [25], and mice were subsequently administered PT (500 mg/kg/day) in 200 μL 5% β-Cyclodextrin by gavage for 2 weeks. Then, they were fed with the same dosage for a further 3 weeks after mice sensitization. To analyze the effects of PT on the epicutaneous test outcomes, four groups of C57BL/6 mice (A, B, C and D; *n* = 5/group) were used. NAC is an antioxidant that is popular due to its ability to minimize oxidative stress. In this study, we compared the effects of NAC (1200 mg/kg/day) with PT for the treatment of Cr(VI)-induced ACD. The detailed administrations are shown in Table 1. For the induction of sensitization, a modification of the coadjuvant chromium sensitization method was used, as described by van Hoogstraten et al. [26]. After a period of 2 weeks, all four of the mice groups were injected intradermally in both inguinal flanks (50 μL each) and ears (20 μL each) with either 0.25% potassium dichromate (dissolved in saline and then emulsified with an equal volume of FCA) or pyrogen-free saline (Control). Fourteen days after sensitization, we boosted mice by injecting 20 μL 0.08% potassium dichromate in saline or pyrogen-free saline (Control) into the pinna of the left ear.

### 2.4. Challenge for Recall and the Epicutaneous Elicitation Test

On day 21 after sensitization, the mice were challenged for recall by painting 20 μL of 2% potassium dichromate (dissolved in 40% DMSO) onto the right ear. As a negative control, 20 μL of 40% DMSO was painted onto the left ear. Delayed-type hypersensitivity reactions were quantified by measuring the increase in ear thickness at 24, 48 and 72 h after the challenge. Measurements were performed using a precision digital thickness caliper from Mitutoya (Digimatic Caliper CD-6” CSX). Swelling and erythema were also documented by photography and by hematoxylin and eosin (H&E) staining of the skin sections of ears that had been collected 72 h after the challenge.

### 2.5. Immunohistochemical (IHC) Staining Analysis

Paraffin-embedded skin tissue sections (5 μM) were dried, deparaffinized, and rehydrated. The hydrated tissue sections were steamed in citrate buffer (pH 6.0; for antigen retrieval), treated with 3% hydrogen peroxide, and blocked with a DAKO antibody diluent (DAKO Corp, Carpinteria, CA, USA) for 1 h at room temperature. The tissue sections were incubated with aliquots of antibodies (Santa Cruz Biotechnology, Santa Cruz, CA, USA) against TNF-α (1:500) and IL-1β (1:100) at 4 °C overnight. After washing, aliquots of biotinylated secondary antibodies (Biotinylated Link Universal) were added. Color development was performed using a labeled streptavidin biotin plus horseradish peroxidase kit (DAKO Corp, Carpinteria, CA, USA). Finally, the slides were counterstained using hematoxylin and examined under light microscopy.

### 2.6. Cells Culture

HaCaT cells were a gift from Professor Hamm-Ming Sheu (Department of Dermatology, College of Medicine, National Cheng Kung University, Taiwan). Cells were cultured in Dulbecco’s Modified Eagle Medium (DMEM) (GIBCO, Grand Island, NY, USA) supplemented with antibiotics, including 100 IU/mL penicillin and 1000 μg/mL streptomycin (Life Technology, Grand Island, NY, USA), and 10% heat-inactivated fetal calf serum (HyClone, South Logan, UT, USA). Cells were maintained under standard growth conditions in a humidified incubator (37 °C and 5% CO_2_.). Exponentially growing cells were detached by 0.1% trypsin-EDTA (Gibco) in phosphate buffered saline (PBS). For the treatment of PT, 100 mM stock solution (in DMSO) was added to the culture medium in a concentrated form, gently mixed for 1 h and treated with different concentrations of Cr(VI). 9 mM Cr(VI) stock solution was prepared inultra pure water (Milli-Q, MQ; Millipore). The cultures were then incubated for different periods of time, as indicated in the figures.

### 2.7. MTT Cell Viability Assay

HaCaT cells were placed in each of the 96 wells (2 × 10^4^/well) and treated with PT and different concentrations of Cr(VI) for 24 h. We prepared stock solutions of 100 mM PT (in DMSO) and 9 mM Cr(VI) (in MQ). Then, 100 μL of 0.5 mg/mL MTT was added, and finally, the solution was incubated at 37 °C for 1 h. After incubation, the MTT was removed and DMSO was added to dissolve the formazan. The optical density was measured by an ELISA reader (Emax, Molecular Devices, Sunnyvale, CA, USA) at 570 nm and the amount of formazan generated was calculated.

### 2.8. Cell Cycle Assay

After treatment, cells were washed with ice-cold PBS, resuspended by trypsinization and fixed with 75% ethanol for at least 16 h. After fixation, the cells were washed with PBS and stained with 50 μg/mL propidium iodide (PI) for 30 min. PI immunofluorescence was measured by flow cytometry (FACScan, Becton Dickinson, San Jose, CA, USA) and the population of nuclei in each phase of the cell cycle was determined using Cell Quest and analyzed by FlowJo 7.6.1. software (Tree Star Inc., Ashland, OR, USA).

### 2.9. Determination of Apoptosis Using Annexin V Staining

Cells were pretreated with PT (20 μM) and/or exposed to Cr(VI). After 24 h of exposure, cells were trypsinized and resuspended in 100 μL of 1 × Annexin V-binding buffer (10 mM hydroxyethyl piperazineethanesulfonic acid (HEPES) (pH 7.4), 0.14 M NaCl and 2.5 mM CaCl_2_) that contained 5 μL of Annexin V-FITC (Becton Dickinson, San Jose, CA, USA) and were incubated at room temperature for 15 min. The 1 × binding buffer (400 μL) was added to stop the reaction, and the stained cells were collected for flow cytometry (FACScalibur, Becton Dickinson, San Jose, CA, USA) analyses. All these data were collected from 10,000 cells per sample and analyzed by FlowJo 7.6.1. software.

### 2.10. ROS Analysis

HaCaT cells were exposed to PT (20 μM) for 1 h before treatment with Cr(VI) (45 μM). After 30, 60 and 90 min of exposure, the level of intracellular ROS generation was estimated using 2,7-dichlorofluorescein diacetate (DCFH-DA, Sigma–Aldrich) dye for cytosolic ROS. Cells were respectively treated with 20 μM DCF-DA incubated at 37 °C for 30 min and washed with PBS. Ultimately, cells were resuspended in staining media and analyzed by flow cytometry (FACScalibur, Becton Dickinson, San Jose, CA, USA). All these data were collected from 10,000 cells per sample. Histograms were obtained from analyses using FlowJo 7.6.1. software and were compared with the histograms of untreated control cells.

### 2.11. 2,2-Diphenyl-1-Picrylhydrazyl (DPPH) Radical Scavenging Activity

Stock solutions of PT (10 mM) were diluted to final concentrations of 10, 20, 50, 150, and 200 μM in ethanol. One hundred and sixty microlitre of 0.1 mM DPPH in ethanol was added to 20 μL of PT, or vitamin C (as a positive control), and the material mixed with 20 μL of water. The mixture was incubated at room temperature for 30 min in the dark. Absorbance at 517 nm was measured on a SpectraMax 340PC384 (Molecular Devices, San Jose, CA, USA). Control was performed in the absence of PT. The percentage of inhibition DPPH% was calculated as follows:

% DPPH inhibition = (DPPH) experiment/(DPPH) control × 100%(1)

All tests were performed in triplicate and inhibition percentages reported as means ± standard deviation (SD).

### 2.12. ER Stress Measurements

The detection of ER stress can be achieved using ER-Tracker Blue-White DPX, a photostable probe that is selective for the endoplasmic reticulum (ER) in live cells [27]. The HaCaT cells were treated with Cr(VI) or PT, then after exposure, the medium was removed from the culture dish, the cells were rinsed with PBS, and prewarmed staining solution was added. The cells were incubated for 30 min at 37 °C, then the loading solution was removed and the cells were washed with PBS. Microscopic images were obtained using the fluorescence microscope.

### 2.13. Western Blot Analysis

The total protein from cell lysates was prepared by harvesting cells in protein extraction buffer for 1 h at 4 °C, as described previously [12]. Lysates were resolved in 10% or 12% Tris-glycine gradient gels and then transferred to the polyvinylidene difluoride (PVDF) membrane. The antibodies for detecting anti-poly (ADP-ribose) polymerase (PARP) antibody were obtained from Millipore (Billerica, MA, USA); phospho-p65, phospho-p38, and IL-1β were purchased from Cell Signaling (Beverly, MA, USA); MK2, GAPDH, *anti*-pro-caspase 1, *anti*-cleaved-caspase 1 antibodies, *anti*-caspase-3 and *anti*-cleaved-caspase-3 antibodies were obtained from Abcam Inc. (Cambridge, MA, USA). *Anti*-NLRP3 was obtained from Adipogen (San Diego, CA, USA). Horseradish peroxidase (HRP)-conjugated *anti*-mouse and *anti*-rabbit secondary antibodies were purchased from Jackson ImmunoResearch Laboratories Inc. (West Grove, PA, USA). TNF-α antibodies were purchased from Abclonal (Woburn, MA, USA). The densities of the bands were quantified with a computer densitometer (AlphaImager 2200 System Alpha Innotech Corporation, San Leandro, CA, USA).

### 2.14. Real-Time Quantitative Polymerase Chain Reaction (qPCR)

HaCaT cells were exposed to PT (20 μM) for 1 h before treatment with Cr(VI) (30 μM). After 12 h of exposure, the RNA was isolated using TRIzol Reagent according to the manufacturer’s instructions (Invitrogen, Carlsbad, CA, USA). RNA concentrations and 260/280 ratios were measured by NanoDrop ND-1000 (Thermo Scientific™, Waltham, MA, USA); the absorbance ratio was ~2.0 for each sample. Reverse transcription (RT) was performed using Superscript II (Life Technologies Inc., Carlsbad, CA, USA). Oligonucleotide primers that correspond to human TNF-α, IL-1β and β-actin were purchased from Stratagene (La Jolla, CA, USA). After the creation of cDNA, the qPCR reactions were performed using a Fast SYBR Green^®^ Master mix and a StepOnePlus™ Real-Time PCR System (Applied Biosystems, Foster, CA, USA) according to the manufacturer’s instructions. Briefly, the qPCR protocol was as follows: 95 °C for 20 s, 40 cycles at 95 °C for 3 s, and finally 58–60 °C for 30 s. The relative expression levels of the genes in each sample were calculated using StepOne software (Version 2.3) and normalized to a housekeeping control (β-actin). The sequences of forward primers and reverse primers were summarized as follows: TNF-α: forward 5′-GCCGCATCGCCGTCTCCTAC - 3′ and reverse 5′-AGCGCTGAGTCGGTCACCC-3′; IL-1β: forward 5′-CAGCTACGAATCTCCGACCAC-3′ and reverse 5′-GGAAGGGAACCAGCATCTTC- 3′; and β-actin: forward 5′-AAGAGAGGCATCCTCACCCT-3′ and reverse 5′-TACATGGCTGGGGTGTTGAA-3′.

### 2.15. Statistical Analysis

All data are presented as means ± SD. Statistical significance was determined using Student’s *t*-test for comparisons between the means or using a one-way analysis of variance with a post hoc Dunnett’s test. We performed all statistical analyses using SPSS 17.0 statistical software (SPSS Inc., Chicago, IL, USA). All statistical tests were performed at a two-sided significance level of 0.05. Images are representative of three or more experiments.

## 3. Results

### 3.1. Pterostilbene (PT) Prevented Hexavalent Chromium (Cr(VI))-Induced Allergic Contact Dermatitis

**Figure 1:**
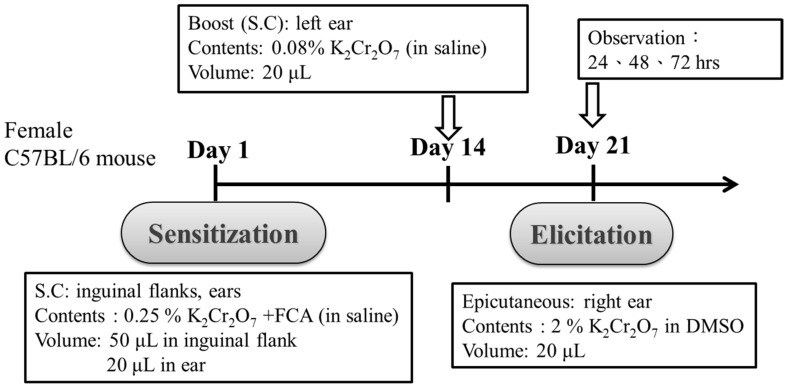
Scheme of intradermal sensitization and epicutaneous elicitation in an allergic contact dermatitis mouse model. S.C.: subcutaneous injection.

**Figure 2:**
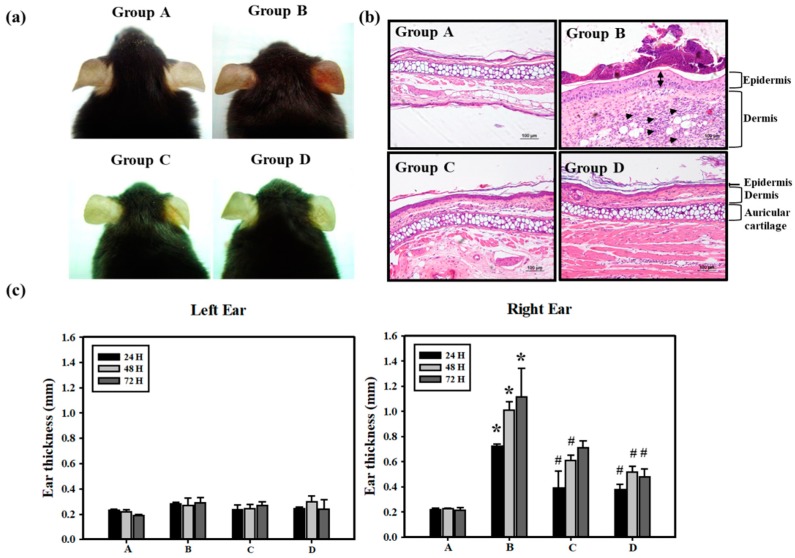
Protective effects of PT on Cr(VI)-induced ACD mice. (**a**) Swelling and erythema were documented by photography at 48 h. (**b**) H&E histology of mice ear skin after the elicitation test with Cr(VI) showed ear swelling and inflammatory cell infiltration at 72 h. In the dermis, the increased number of inflammatory cells, neutrophils, was observed (arrowhead) in K_2_Cr_2_O_7_ treatment (Group B). In the epidermis, Group B showed the stratum corneum thickening (bi-arrowhead), but NAC (Group C) or PT (Group D) treatment significantly suppressed Cr(VI)-induced increases in ear thickness. Also, no differences in the auricular cartilage were observed (scale bar representing 100 μM). (**c**) ACD reactions were quantified by measuring the ear thickness at 24, 48 and 72 h after the elicitation test (*n* = 5). The time course measurement of Left (vehicle) and Right (epicutaneous) ear thickness. (A: Control; B: K_2_Cr_2_O_7_; C: NAC; D: PT) (* *p* <0.05 versus control group; # *p* <0.05 versus K_2_Cr_2_O_7_ group).

**Figure 3:**
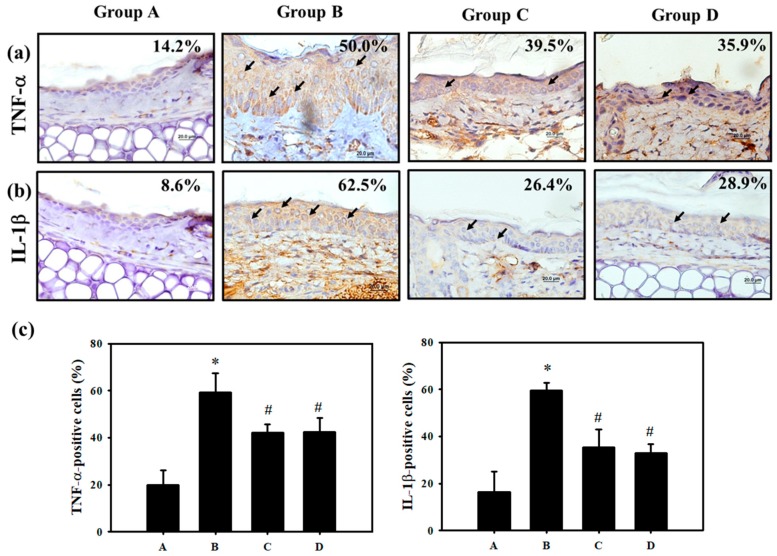
PT protects against pro-inflammatory cytokines in mice with ACD induced by Cr(VI). Skin biopsy from the Control, K_2_Cr_2_O_7_, NAC and PT groups shows immunohistochemical staining for analysis of (**a**) TNF-α. (**b**) IL-1β- positive cells (brown) in the epidermis (scale bar representing 20 μM). (**c**) The quantification of TNF-α and IL-1β-positive cells was determined using HistoQuest software (Version 4.0.4.0158, TissueGnostics). (*n* = 3, * *p* <0.05 versus control group; # *p* <0.05 versus K_2_Cr_2_O_7_ group).

To induce allergic contact dermatitis (ACD), the mice were sensitized by Cr(VI)/Freund’s complete adjuvant (FCA)–chromium emulsion on day 1 , and then rechallenged with Cr(VI) alone on Day 14. We recorded the swelling and erythema of mice ear skin after epicutaneous tests at 24, 48 and 72 h (Figure 1). Clinically, it usually takes months to years for cement workers to become sensitized to Cr(VI) [28], and it is difficult to observe the effects of preventative agents that might benefit cement workers in a short period of time. FCA injections generally yield the best ACD response for metals, such as nickel and chromium [26]. In the present study, we effectively induce chromium hypersensitivity within 3 weeks, with a mixture of FCA and Cr(VI). The application procedure is shown in Table 1. In Groups C and D, the mice were administered PT (500 mg/kg/day) and NAC (1200 mg/kg/day) during the entire experiment. As illustrated in Figure 2, Cr(VI) solution produced cutaneous inflammation in the mice’s right ear. The skin reaction was accompanied by a prominent erythema (Figure 2a, Group B), and the increase in ear thickness was associated with characteristic pathological features of keratinocyte impairment and inflammatory cell infiltration (Figure 2b,c, Group B). In contrast, the formation of ear erythema (Figure 2a, Group C and D), inflammatory cell infiltration (Figure 2b, Group C and D), and ear thickness (Figure 2c, Group C and D) post-challenge was attenuated when mice were treated with PT and NAC. Basically, only a lower dose of PT is needed in mice to achieve the same effect as feeding NAC. In the mice skin biopsy, the results indicated that Cr(VI) significantly elevated TNF-α (50.0%) and IL-1β (62.5%) expression at the protein level by immunohistochemistry staining. PT could significantly suppress the inflammatory response in the epidermis and inhibit TNF-α and IL-1β expression (Figure 3).

### 3.2. Cr(VI)-Triggered Apoptosis was Eliminated by PT in HaCaT Cells

**Figure 4:**
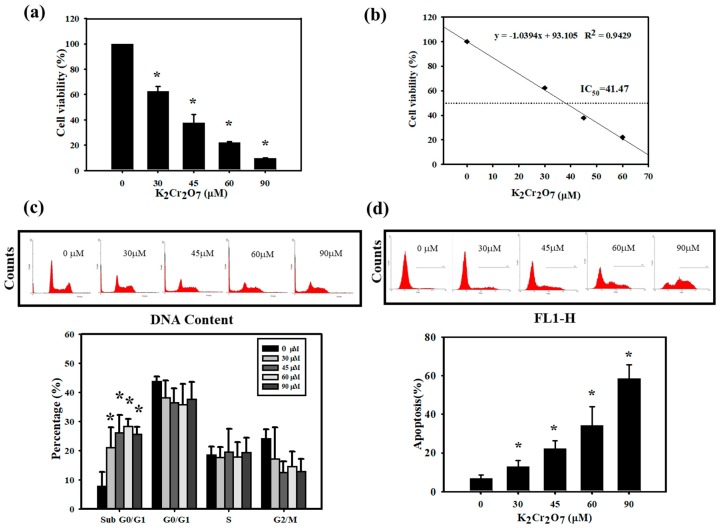
Measurement of cell viability, cell cycle distribution and apoptosis of Cr(VI) in HaCaT cells. The cells were treated with 0, 30, 45, 60 or 90 μM Cr(VI) for 24 h. (**a**) Cell viability was measured using the MTT assay. Cr(VI) decreased the viability of HaCaT cells in a concentration-dependent manner. (**b**) Determination of Cr(VI) IC_50_ values by linear regression. (**c**) Cells were collected and incubated with 50 μg/mL of PI for 30 min and subjected to flow cytometry analysis to examine the cell distribution at each phase of the cell cycle. The percentage of cells in the sub G0/G1 phase were significantly increased when cells were exposed to Cr(VI). (**d**) Early apoptosis detection was measured by an Annexin-V apoptosis detection kit. Flow cytometry analysis indicating the rate of apoptosis in HaCaT cells treated with Cr(VI). Quantification of early apoptosis in HaCaT cells exposed to Cr(VI) in a concentration-dependent manner. All above data are presented as the mean ± standard deviation (SD) from three independent experiments. * *p* <0.05 versus untreated cells.

**Figure 5:**
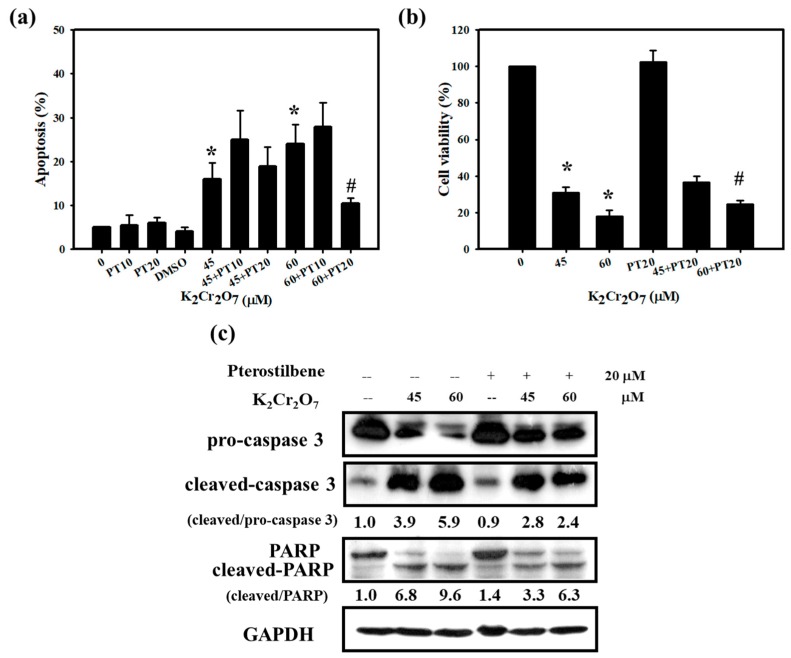
Pretreatment with PT rescued Cr(VI)-induced cytotoxicity. (**a**) HaCaT cells were pretreated with 10 or 20 μM PT for 1 h. Early apoptosis, detected using an Annexin V apoptosis detection kit, showed that PT (20 μM) inhibited Cr(VI)-induced apoptosis at different concentrations of Cr(VI) (45 and 60 μM) exposure for 24 h. (**b**) Cell viability was measured using MTT assay. HaCaT cells exposed to Cr(VI) for 24 h after pretreatment with 20 μM PT. PT slightly increased the cell viability of Cr(VI) (60 μM). Data are presented as the mean ± SD from three independent experiments. * *p* <0.05 versus control; # *p* <0.05 versus the Cr(VI)-treated group. (**c**) The expression of apoptosis proteins, pro-caspase 3, cleaved-caspase 3, PARP and GAPDH were monitored after 24 h of Cr(VI) or Cr(VI) plus PT exposure in HaCaT cells. Western blots are representative of *n* = 3 experiments.

Since we found that PT attenuated skin inflammation and the ACD response induced by Cr(VI), we carried out experiments in vitro to explore the underlying mechanisms for the attenuation of skin inflammation by PT. It is well known that the induction of keratinocyte damage plays a critical role in cutaneous inflammation [17]. We treated HaCaT cells with different concentrations of Cr(VI) for 24 h. Cytotoxicity was determined using MTT assays. As shown in Figure 4a, Cr(VI) (ranging from 30 to 90 μM) significantly reduced the number of viable cells and the half maximal inhibitory concentrations (IC_50_) value of Cr(VI) was 41.47 μM at 24 h of treatment (Figure 4b). The toxicity effect was also confirmed with the influence of Cr(VI) on cell cycle progression. The cell cycle distribution was measured via flow cytometry. The percentages of cells in the sub G0/G1 were observed after different concentrations of Cr(VI) (30 to 90 μM) treatment for 24 h (Figure 4c). Generally, the cell cycle is tightly regulated to maintain the integrity of the cell, and any cell that is defective or unable to complete the cell cycle is programmed to die by apoptosis [29]. Because apoptotic cells have reduced DNA content compared with living cells, we could observe a large amount of sub-G0/G1 accumulation when cells were treated with Cr(VI). On the other hand, we also quantified the ratio of apoptosis with annexin-V staining by flow cytometry. As illustrated in Figure 4d, Cr(VI) resulted in concentration-dependent toxicity and apoptotic cell death. The half maximal effective concentration (EC_50_) value for apoptosis was 84.30 µM and shown with a graph linear regression in Figure S2. To confirm whether PT could inhibit the death of keratinocytes, we further measured cell viability and apoptosis when HaCaT cells were treated with Cr(VI) and PT alone or in combination. As expected, PT (20 μM) significantly protected cells against 60 μM Cr(VI)-induced apoptosis and cell death but no significant changes at lower PT concentration (10 μM) (Figure 5a,b). Similarly, caspase 3 and poly(ADP-ribose) polymerase(PARP) protein expression verified the same trend of changes for PT protective effects. Caspase-3 activation and the induction of PARP cleavage are considered to be hallmarks of apoptosis [30,31]. Cr(VI) markedly induced cleaved-caspase 3 and cleaved-PARP protein expression, but these proteins were downregulated by PT pretreatment (Figure 5c). The findings indicated that PT has the ability to rescue cell death by attenuating the activation of apoptotic-related proteins.

### 3.3. Cr(VI) Treatment Induced Reactive Oxygen Species (ROS) Generation and Endoplasmic Reticulum (ER) Stress in HaCaT Cells, Which were Attenuated by PT

**Figure 6:**
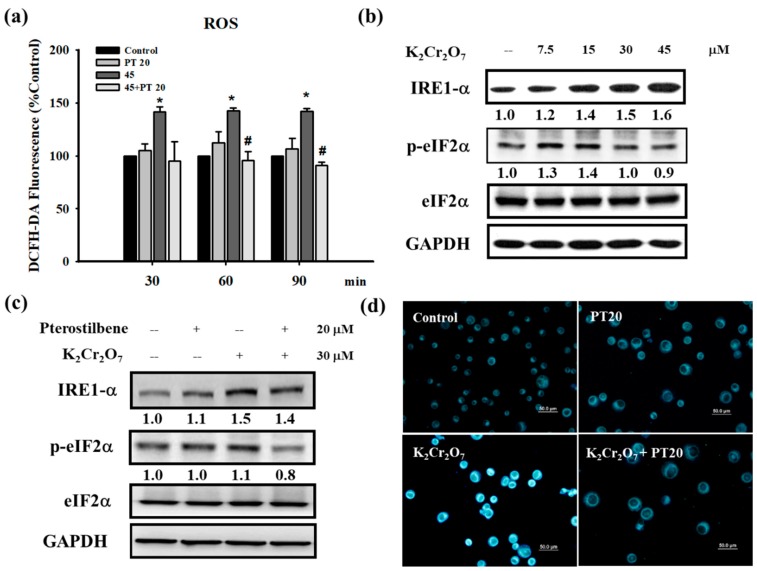
Cr(VI) treatment induced ROS and ER stress in HaCaT cells, which were inhibited by PT. (**a**) HaCaT cells were treated with 45 μM of Cr(VI) for various time periods (30, 60, and 90 min). The amount of ROS produced in cells was determined using the fluorescence dye (DCFH-DA) and analyzed by flow cytometry. Pretreatment with PT (20 μM) could suppress ROS generation. Fluorescence units reflected the subtraction of untreated cell values and were presented as the mean ± SD from three independent experiments. * *p* <0.05 versus control; # *p* <0.05 versus the Cr(VI)-treated group. (**b**) Cell lysates extracted from HaCaT cells treated with several concentrations (7.5, 15, 30, and 45 μM) of Cr(VI) for 24 h were submitted to Western blot analysis to detect the protein expression of ER stress. Cr(VI) upregulated IRE1α and p-eIF2α protein expressions but p-eIF2α decreased at 45 μM of Cr(VI). (**c**) HaCaT cells were treated with 30 μM of Cr(VI) for 24 h. Pretreatment with PT (20 μM) inhibited ER stress-related protein expression. Western blots are representative of *n* = 3 experiments. (**d**) HaCaT cells were treated with Cr(VI) (30 μM) for 24 h. The strength of the ER tracker was enhanced by Cr(VI) and alleviated by PT pretreatment. The scale bars are 50 μM.

The production of free radicals and ROS is one commonly proposed toxicological mechanism of toxic compounds. Cr(VI) is reduced to its trivalent form in the cells, and most studies indicate that free radicals and ROS that are produced in this process result in cell damage and the inflammatory response [32,33,34]. In our study, an elevated level of ROS was observed when HaCaT cells were treated with Cr(VI) (45 μM) at 30, 60, and 90 min. PT pretreatment significantly inhibited ROS generation at 60 and 90 min (Figure 6a). In addition, the antioxidant activity of PT was estimated by performing its free radical scavenging activity at different concentrations (10, 20, 50, 150, and 200 μM). In the DPPH radical scavenging assay, PT exhibited concentration-dependent activity from 10 μM to 200 μM (Figure S3). The results proved that, as an antioxidant, PT did act as a free radical scavenger. Previous research has shown that Cr(VI) induces cytotoxicity through the activation of ROS-mediated ER stress in hepatocytes [13]. We examined the main ER-stress signaling proteins, IRE1α (inositol-requiring 1α) and eukaryotic translation-initiation factor 2α (eIF2α) whose phosphorylation is regulated by PERK (double-stranded RNA-dependent protein kinase (PKR)-like ER kinase), to determine whether an increase of ROS by Cr(VI) triggered ER stress in HaCaT cells. As shown in Figure 6b, the expression of IRE1α and p-eIF2α in the cells treated with Cr(VI) increased when compared with those in the control cells. The total protein expression of eIF2α did not change in Cr(VI) treatment. However, the phosphorylation of the eIF2α protein decreased with exposure to higher concentrations of Cr(VI). Higher concentrations of Cr(VI) might inhibit p-eIF2α protein expression by the induction of cellular apoptosis, particularly the activation of caspase 3/7 [35]. We also evaluated the level of IRE1α and p-eIF2α in HaCaT cells after treatment with Cr(VI) and PT. HaCaT cells were treated by 30 μM of Cr(VI) for 24 h with or without 20 μM PT pretreatment for 1 h, followed by Western blot analysis. As shown in Figure 6c, the levels of IRE1α and p-eIF2α protein were significantly decreased after PT pretreatment. In the fluorescence staining test, Cr(VI) (30 μM) significantly increased the fluorescence staining intensity, representing the remarkable induction of ER stress. Similar to the expression of ER stress proteins, PT (20 μM) suppressed the induction of ER stress fluorescence staining intensity by Cr(VI) (Figure 6d).

### 3.4. PT Inhibited Pro-Inflammatory Cytokines Production by Regulating p38 MAPK/ MK2 Pathway and the NLRP3-Inflammasome

**Figure 7:**
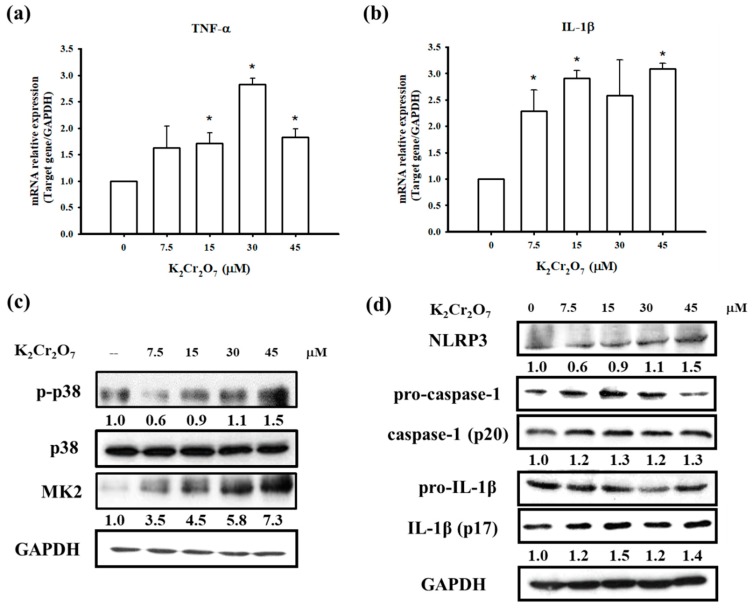

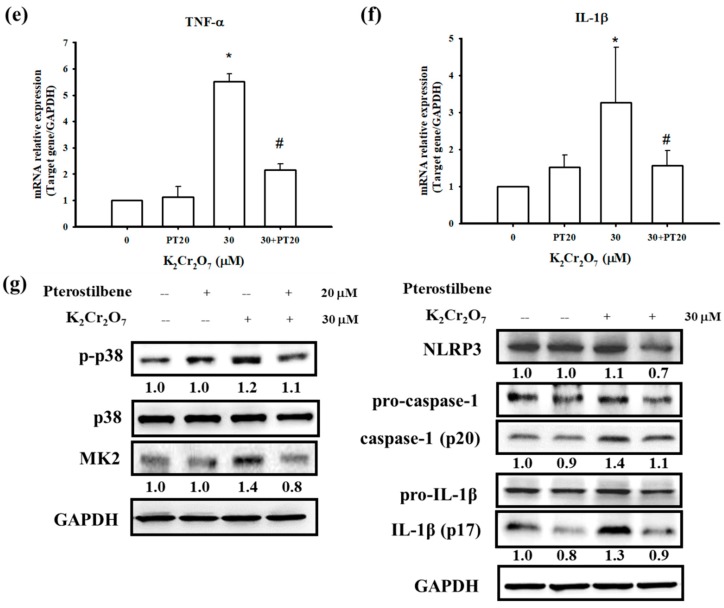
PT attenuates the Cr(VI)-induced pro-inflammatory cytokines and NLRP3 inflammasome. (**a**,**b**) The mRNA expressions of TNF-α and IL-1β were measured by real-time PCR. HaCaT cells were treated with several concentrations (7.5, 15, 30, and 45 μM) of Cr(VI) for 12 h. Data presented as the mean ± SD from three independent experiments. * *p* <0.05 versus controls. (**c**,**d**) The expression of the p38 MAPK/MK2 signaling pathway and NLRP3 inflammasome-associated proteins were measured by Western blot analysis following treatment with several concentrations of Cr(VI) for 24 h. (**e**,**f**) The cells were pretreated with PT (20 μM) for 1 h and then treated with Cr(VI) (30 μM) for an additional 12 h. Real-time PCR showed that the mRNA levels of pro-inflammatory cytokines, TNF-α and IL-1β decreased after treatment with PT. (**g**) Western blot analysis demonstrated that the activation of the p38 MAPK/MK2 signaling pathway and the NLRP3 inflammasome was suppressed by PT. Western blots are representative of *n* = 3 experiments.

ACD is an inflammatory skin disease in which the release of pro-inflammatory cytokines is involved in its development. The p38 MAPK/MK2 signaling pathway is a major regulator of stress- and cytokine-induced gene expression at the transcriptional and post-transcriptional level [36]. The role of MK2 has also been explored in an inflammatory disorders of the skin. MK2-deficient mice exhibited a significant reduction in the level of pro-inflammatory cytokines, such as TNF-α, IL-1β and IFN-γ, in skin after oxazolone challenge [37]. In our study, we attempted to identify whether Cr(VI) resulted in the production of pro-inflammatory cytokines via the p38 MAPK/MK2 pathway. As shown in Figure 7a,b, the mRNA expression of TNF-α and IL-1β was significantly increased by Cr(VI) treatment in HaCaT cells. At the same time, we also observed the concentration-dependent expression of p-p38 and MK2 proteins (Figure 7c). Our previous studies have indicated that Cr(VI) can activate p38 MAPK protein expression, and this study further found that the downstream protein MK2 of p38 MAPK can also be activated by Cr(VI). On the other hand, we analyzed the involvement of the NLRP3 inflammasome which is a large IL-1β activating platform, which assembles in response to extracellular and intracellular stimuli and leads to the activation of caspase-1 [38]. Western blot analyses showed Cr(VI) markedly induced NLRP3, Caspase-1 (p20), and IL-1β (p17) protein expressions (Figure 7d). Our results were similar to a recent study that investigated the capacity of allergic contact dermatitis-relevant Cr(VI) compounds to trigger cellular innate immune activation and showed that Cr(VI) is a potent NLRP3 inflammasome activator [39]. Next, we explored the role of PT in the Cr(VI) induced inflammatory response. Comparison of TNF-α and IL-1β mRNA expression from pretreatment with or without 20 μM PT showed that PT abolished cytokine expressions by Cr(VI) (Figure 7e,f). Further it compromised the Cr(VI) induced p38 MAPK/MK2 signaling pathway and NLRP3 inflammasome activation, as indicated by absence of p-p38, MK2, cleaved caspase-1 (p20) and IL-1β (p17) proteins of PT-exposed cells (Figure 7g). These results suggest that PT not only attenuated intracellular ROS induced by Cr(VI) but was also capable of regulating TNF-α and IL-1β expression via the p38 MAPK/MK2 pathway and the NLRP3 inflammasome.

## 4. Discussion

Most exposure to chromium occurs in occupational settings, especially in industrial processes, such as cement containing chromium salts in the construction industry [40]. Approximately one third of bricklayers and stonemasons who are in frequent contact with cement receive a diagnosis of Cr(VI) skin disease. Chromium causes irritant contact dermatitis and allergic contact dermatitis [41]. The inflammatory response is regulated by many signaling pathways. One of the mechanisms is the MAPK pathway. In our previous studies, we found that Cr(VI) increased ROS formation and activated MAPK pathways, including extracellular-signal-regulated kinase (ERK), c-Jun *N*-terminal kinase (JNK) and p38 [11,12]. Previous research has shown that MK2, a substrate of p38 MAPK, contributes to inflammation due to its essential role in promoting the expression of TNF-α, IL-1β, and IL-6 in macrophages [42]. In the current study, Cr(VI) increased TNF-α, p-p38 and MK2 in HaCaT cells (Figure 7a,c). Furthermore, PT inhibited Cr(VI)-induced TNF-α, p-p38 and MK2 (Figure 7e,g). The accumulated evidence shows that Cr(VI) leads to ROS accumulation, which results in potassium (K^+^) efflux, thereby activating the NLRP3 inflammasome. The processes are sequential: first ROS generation, then K^+^ efflux, followed by NLRP3 activation and IL-1β release [43,44]. Evidence is presented indicating that strong sensitizers and irritants trigger inflammasome activation, and it seems that inflammasome activation is a common feature of sensitizing haptens [45]. Brauer et al. indicated that NAC, which is a well-known ROS scavenger, eliminated the Cr(VI)-induced mitochondrial ROS [46]. In our previous study, we used NAC to evaluate its effects on coadjuvant chromium-sensitized albino guinea pigs, and found that NAC (1200 mg/kg/day) significantly diminished chromium hypersensitivity by counteracting the formation and effects of ROS. Therefore, we used the same dosage of NAC as a comparing group to determinate the ability of PT on inhibition of coadjuvant chromium-sensitized mice.

In our pilot study, oral administration of 250 mg/kg/day and 500 mg/kg/day of PT were used to evaluate its effects on chromium hypersensitivity (data not show). Only 500 mg/kg/day of PT group showed a significant decrease of the sensitization rate of chromium hypersensitivity. As a dietary phytophenol, pterostilbene appeared to be very safe. Recent studies in human and in animal models have investigated the safety profile of PT and suggest that PT does not have significant toxic effects. A long-term and double-blind clinical trial indicates that PT is generally safe for use in humans at doses up to 250 mg per day [47]. Another in vivo study points out that employing PT at 30, 300, and 3000 mg/kg/day (28 days) in mice attains safe status [48]. In addition, PT demonstrates greater bioavailability and total plasma levels of both the parent compound and metabolites than does resveratrol. Studies in rats report that an oral dose of 168 mg/kg of PT maintains a very high serum level (around 10 μg/mL) for over eight hours after administration [49]. The major metabolic routes of PT are glucuronidation, sulfation, hydroxylation and demethylation. Recently, a study has showed that pinostilbene was identified as a major metabolite of PT in CD-1 mouse colonic content and mucosa. The level of pinostilbene was found to be approximately equivalent to that of pterostilbene in the colonic mucosa [50].

Pterostilbene inhibited inflammation and ROS production in chondrocytes by activating the Nrf2 pathway [51]. Our in vivo study further demonstrated that Cr(VI) solution produced cutaneous inflammation in the mouse ear (Figure 2). The skin reaction was accompanied by a prominent erythema and the increase in ear thickness, keratinocyte impairment, and inflammatory cell infiltration. In contrast, PT and NAC attenuated the formation of ear erythema, inflammatory cell infiltration, and ear thickness. In the mouse skin tissues, the results indicated that PT significantly suppressed Cr(VI)-elevated TNF-α and IL-1β expression (Figure 3). Furthermore, PT pretreatment significantly inhibited ROS generation in HaCaT cells (Figure 6a). Previous research has shown that PT attenuated early brain injury via the inhibition of the NLRP3 inflammasome and Nox2-related oxidative stress [52]. We found that PT significantly inhibited the Cr(VI)-induced expression of NLRP3, cleaved-caspase 1 and cleaved-IL-1β (Figure 7f,g).

The basis of Cr(VI) toxicity seems to relate to its strong oxidative capacity, which leads to cell death [43]. More recently, data accumulated by us and others have revealed that Cr(VI) treatments significantly inhibited the cell viability and induced apoptosis [12,13]. Furthermore, HaCaT cells pretreated with NAC exhibited a decrease in apoptosis and affected cell viability [12]. Xie et al. have confirmed that Cr(VI) decreased the mitochondrial membrane potential, leading to apoptosis in hepatocytes [53]. In the present study, we found that Cr(VI) significantly inhibited the viability and induced cell apoptosis in HaCaT cells (Figure 4). Recent evidence shows that PT decreased hypoxia-induced oxidative stress and apoptosis in cardiomyocytes [54]. Another recent study concluded that PT significantly reduced cardiomyocyte apoptosis, increased Bcl-2 and p-Akt levels and decreased Bax expression in a rat model of myocardial ischemia-reperfusion injury [55]. Our current results also showed that PT rescues cell death by preventing Cr(VI)-activated apoptotic-related proteins in keratinocytes (Figure 5).

The global expression of mRNAs in three normal and Cr(VI)-treated hepatocytes has been analyzed using microarrays. Cr(VI) treatment altered the expression of multiple genes involved in a variety of biological processes, including redox, apoptosis, ER stress and the cell cycle. Of note, Cr(VI) significantly induced the expression of ER stress-associated genes, including CHOP, PERK and IRE1 [13]. Our current data are consistent with this study, demonstrating that Cr(VI)-induced ER stress in HaCaT cells (Figure 6b). The initial role of ER stress is an adaptive protective process in eukaryotic cells. However, when ER stress is too severe to recover from, it changes from a pro-survival to a pro-death response [56]. Previous studies have demonstrated that ROS production could induce cell apoptosis via the activation of both the ER stress and mitochondrial pathways [57]. Furthermore, ER stress activates the unfolded protein response (UPR) pathway, which can cause cell apoptosis [56]. Recent evidence showed that the inhibition of ER stress partially prevented Cr(VI)-induced cell apoptosis, mitochondrial dysfunction and ROS generation [13]. Liu et al. indicated that PT treatment reduced the TNF-α-induced effects exerted by ER stress-related molecules. In addition, thapsigargin, an ER stress inducer, attenuated the protective effect of PT against TNF-α-induced inflammation and ER stress in human umbilical vein endothelial cells (HUVECs) [58]. In our current study, PT suppressed the induction of ER stress triggered by Cr(VI) (Figure 6c,d). The multiple benefits of PT in the treatment and prevention of human disease have been attributed to its anti-oxidative and anti-inflammatory effects [20]. For example, PT has been indicated to effectively prevent chronic UVB-induced oxidative damages and inflammation by modulating Nrf2 activity, which has been reported to serve as a central regulator of UV protection in the epidermis [59]. Recent studies indicated that UV radiation can cause DNA damage and the genotoxic stress is a trigger for autophagy, an essential, homeostatic cellular process of “self-eating” [60]. Induction of autophagy is also likely to be regulated by UV-induced p53 and be able to initiate autophagy through negative regulation of mTOR by p53 in response to DNA damage [61]. On the other hand, autophagy has been linked with keratinocyte differentiation as well as with the pathogenesis of diverse skin disorders including, systemic sclerosis, psoriasis, and skin carcinogenesis [62]. PT, as an autophagy activator in our previous study [63], the ability of PT attenuating chromium hypersensitivity by autophagy might be worthy of further in-depth investigation.

## 5. Conclusions

**Figure 8:**
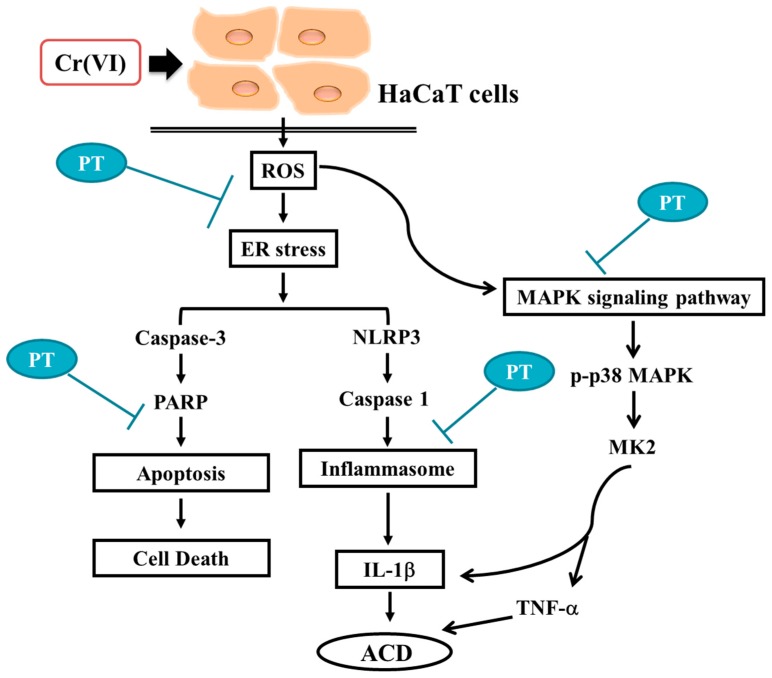
Possible molecular targets of the preventive effects of PT in Cr(VI)-induced ACD. Cr(VI) activated NLRP3 inflammasome activity and the p38 MAPK/MK2 signaling pathway leading to pro-inflammatory gene expression and resulting in keratinocyte cell death by apoptosis. The administration of PT inhibited cell death, the NLRP3 inflammasome and pro-inflammatory cytokines, which were regulated by ROS generation and ER stress and were related to Cr(VI)-induced ACD.

We demonstrated the mechanisms underlying the molecular action of Cr(VI)-induced inflammation and apoptosis in keratinocytes (Figure 8). Furthermore, the results found that PT attenuated Cr(VI) treatment-induced ROS generation and ER stress in HaCaT cells. PT inhibited pro-inflammatory cytokine production by regulating the p38 MAPK/MK2 pathway and NLRP3-inflammasome. In addition, PT eliminated Cr(VI)-triggered apoptosis. Our in vivo evidence demonstrated that PT prevented Cr(VI)-induced allergic contact dermatitis. These findings suggest that PT can be considered to have a potential therapeutic value in the prevention and rescue of Cr(VI) skin disease.

## Figures and Tables

**Table 1:** The feeding regimen, NAC and pterostilbene (PT) administration and application procedure of female C57BL/6 groups for the epicutaneous test.

Group	Feeding Regimen and Antioxidants Administration	Application Procedure	Epicutaneous Test
A (Control)	Normal diet	Injected with saline	21 days after injection treatment
B (K_2_Cr_2_O_7_)	Normal diet and continually gave 5% β-Cyclodextrin (200 μL/day) by gavage until the end of the experiment	Injected with K_2_Cr_2_O_7_ in FCA emulsion	21 days after injection treatment
C (NAC)	NAC 1200 mg/kg/day for 2 weeks, and then fed with the same dosage for a further 3 weeks after injection treatment	Injected with K_2_Cr_2_O_7_ in FCA emulsion	21 days after injection treatment
D (PT)	PT 500 mg/kg/day for 2 weeks, and then fed with the same dosage for a further 3 weeks after injection treatment	Injected with K_2_Cr_2_O_7_ in FCA emulsion	21 days after injection treatment

## Supplementary Materials

The following are available online at http://www.mdpi.com/2077-0383/7/12/489/s1, Figure S1: Chemical structure of *trans*-3,5-dimethoxy-4′-hydroxystilbene (pterostilbene), Figure S2: Percentage of apoptotic cells determined in different Cr(VI) concentrations. The EC50 value for apoptosis was 84.303 µM. Data presented as the mean ± standard deviation (SD) from three independent experiments, Figure S3: DPPH radical scavenging activity of PT. PT antioxidant activity was assessed via the percent inhibition (radical scavenging) of DPPH. Vitamin C (200 μM) was used as a positive control. All tests were performed in triplicate and results are expressed as means ± SD.

## References

[1] Shelnutt S.R., Goad P., Belsito D.V. (2007). Dermatological toxicity of hexavalent chromium. Crit. Rev. Toxicol..

[2] Goh C.L., Gan S.L., Ngui S.J. (1986). Occupational dermatitis in a prefabrication construction factory. Contact Dermat..

[3] Guo Y.L., Wang B.J., Yeh K.C., Wang J.C., Kao H.H., Wang M.T., Shih H.C., Chen C.J. (1999). Dermatoses in cement workers in southern Taiwan. Contact Dermat..

[4] Toncic R.J., Lipozencic J., Martinac I., Greguric S. (2011). Immunology of allergic contact dermatitis. Acta Dermatovenerol. Croat..

[5] Fuchs J., Zollner T.M., Kaufmann R., Podda M. (2001). Redox-modulated pathways in inflammatory skin diseases. Free Radic. Biol. Med..

[6] Matos T.J., Duarte C.B., Goncalo M., Lopes M.C. (2005). Role of oxidative stress in ERK and p38 MAPK activation induced by the chemical sensitizer DNFB in a fetal skin dendritic cell line. Immunol. Cell Biol..

[7] Chao W., Deng J.-S., Huang S.-S., Li P.-Y., Liang Y.-C., Huang G.-J. (2017). 3,4-dihydroxybenzalacetone attenuates lipopolysaccharide-induced inflammation in acute lung injury via down-regulation of MMP-2 and MMP-9 activities through suppressing ROS-mediated MAPK and PI3K/AKT signaling pathways. Int. Immunopharmacol..

[8] Singh R.K., Najmi A.K., Dastidar S.G. (2017). Biological functions and role of mitogen-activated protein kinase activated protein kinase 2 (MK2) in inflammatory diseases. Pharmacol. Rep..

[9] Chen R.J., Lee Y.H., Yeh Y.L., Wang Y.J., Wang B.J. (2016). The Roles of Autophagy and the Inflammasome during Environmental Stress-Triggered Skin Inflammation. Int. J. Mol. Sci..

[10] Corsini E., Galbiati V., Nikitovic D., Tsatsakis A.M. (2013). Role of oxidative stress in chemical allergens induced skin cells activation. Food Chem. Toxicol..

[11] Wang B.J., Sheu H.M., Guo Y.L., Lee Y.H., Lai C.S., Pan M.H., Wang Y.J. (2010). Hexavalent chromium induced ROS formation, Akt, NF-kappaB, and MAPK activation, and TNF-alpha and IL-1alpha production in keratinocytes. Toxicol. Lett..

[12] Lee Y.H., Su S.B., Huang C.C., Sheu H.M., Tsai J.C., Lin C.H., Wang Y.J., Wang B.J. (2014). *N*-acetylcysteine attenuates hexavalent chromium-induced hypersensitivity through inhibition of cell death, ROS-related signaling and cytokine expression. PLoS ONE.

[13] Zhang Y., Xiao F., Liu X., Liu K., Zhou X., Zhong C. (2017). Cr(VI) induces cytotoxicity in vitro through activation of ROS-mediated endoplasmic reticulum stress and mitochondrial dysfunction via the PI3K/Akt signaling pathway. Toxicol. In Vitro.

[14] Zhang K., Kaufman R.J. (2008). From endoplasmic-reticulum stress to the inflammatory response. Nature.

[15] Lee S.E., Takagi Y., Nishizaka T., Baek J.H., Kim H.J., Lee S.H. (2017). Subclinical cutaneous inflammation remained after permeability barrier disruption enhances UV sensitivity by altering ER stress responses and topical pseudoceramide prevents them. Arch. Dermatol. Res..

[16] Sano R., Reed J.C. (2013). ER stress-induced cell death mechanisms. Biochim. Biophys. Acta (BBA)—Mol. Cell Res..

[17] Raj D., Brash D.E., Grossman D. (2006). Keratinocyte apoptosis in epidermal development and disease. J. Investig. Dermatol..

[18] Osorio F., Lambrecht B.N., Janssens S. (2018). Antigen presentation unfolded: Identifying convergence points between the UPR and antigen presentation pathways. Curr. Opin. Immunol..

[19] Zitvogel L., Kepp O., Kroemer G. (2010). Decoding cell death signals in inflammation and immunity. Cell.

[20] McCormack D., McFadden D. (2013). A review of pterostilbene antioxidant activity and disease modification. Oxid. Med. Cell Longev..

[21] Perecko T., Jancinova V., Drabikova K., Nosal R., Harmatha J. (2008). Structure-efficiency relationship in derivatives of stilbene. Comparison of resveratrol, pinosylvin and pterostilbene. Neuro. Endocrinol. Lett..

[22] Dvorakova M., Landa P. (2017). Anti-inflammatory activity of natural stilbenoids: A review. Pharmacol. Res..

[23] Chen R.J., Lee Y.H., Yeh Y.L., Wu W.S., Ho C.T., Li C.Y., Wang B.J., Wang Y.J. (2017). Autophagy-inducing effect of pterostilbene: A prospective therapeutic/preventive option for skin diseases. J. Food Drug Anal..

[24] Pettit G.R., Grealish M.P., Jung M.K., Hamel E., Pettit R.K., Chapuis J.C., Schmidt J.M. (2002). Antineoplastic agents. 465. Structural modification of resveratrol: Sodium resverastatin phosphate. J. Med. Chem..

[25] Yeo S.C., Ho P.C., Lin H.S. (2013). Pharmacokinetics of pterostilbene in Sprague-Dawley rats: The impacts of aqueous solubility, fasting, dose escalation, and dosing route on bioavailability. Mol. Nutr. Food Res..

[26] van Hoogstraten I.M., de Groot J., Boden D., von Blomberg B.M., Kraal G., Scheper R.J. (1992). Development of a concomitant nickel and chromium sensitization model in the guinea pig. Int. Arch Allergy Immunol..

[27] Abdelrahim M., Newman K., Vanderlaag K., Samudio I., Safe S. (2006). 3,3′-Diindolylmethane (DIM) and its derivatives induce apoptosis in pancreatic cancer cells through endoplasmic reticulum stress-dependent upregulation of DR5. Carcinogenesis.

[28] Avnstorp C. (1992). Cement eczema. An epidemiological intervention study. Acta. Derm. Venereol. Suppl. (Stockh).

[29] Crowley L.C., Chojnowski G., Waterhouse N.J. (2016). Measuring the DNA content of cells in apoptosis and at different cell-cycle stages by propidium iodide staining and flow cytometry. Cold Spring Harb. Protoc..

[30] Chaitanya G.V., Steven A.J., Babu P.P. (2010). PARP-1 cleavage fragments: Signatures of cell-death proteases in neurodegeneration. Cell Commun. Signal..

[31] Kaufmann S.H., Desnoyers S., Ottaviano Y., Davidson N.E., Poirier G.G. (1993). Specific proteolytic cleavage of poly(ADP-ribose) polymerase: An early marker of chemotherapy-induced apoptosis. Cancer Res..

[32] Husain N., Mahmood R. (2017). Hexavalent chromium induces reactive oxygen species and impairs the antioxidant power of human erythrocytes and lymphocytes: Decreased metal reducing and free radical quenching ability of the cells. Toxicol. Ind. Health.

[33] Wang B.J., Guo Y.L., Chang H.Y., Sheu H.M., Pan M.H., Lee Y.H., Wang Y.J. (2010). N-acetylcysteine inhibits chromium hypersensitivity in coadjuvant chromium-sensitized albino guinea pigs by suppressing the effects of reactive oxygen species. Exp. Dermatol..

[34] Jomova K., Valko M. (2011). Advances in metal-induced oxidative stress and human disease. Toxicology.

[35] Schleicher S.M., Moretti L., Varki V., Lu B. (2010). Progress in the unraveling of the endoplasmic reticulum stress/autophagy pathway and cancer: Implications for future therapeutic approaches. Drug Resist. Updates.

[36] Menon M.B., Tiedje C., Lafera J., Ronkina N., Konen T., Kotlyarov A., Gaestel M. (2013). Endoplasmic reticulum-associated ubiquitin-conjugating enzyme Ube2j1 is a novel substrate of MK2 (MAPKAP kinase-2) involved in MK2-mediated TNFalpha production. Biochem. J..

[37] Funding A.T., Johansen C., Gaestel M., Bibby B.M., Lilleholt L.L., Kragballe K., Iversen L. (2009). Reduced oxazolone-induced skin inflammation in MAPKAP kinase 2 knockout mice. J. Investig. Dermatol..

[38] Menu P., Vince J.E. (2011). The NLRP3 inflammasome in health and disease: The good, the bad and the ugly. Clin. Exp. Immunol..

[39] Adam C., Wohlfarth J., Haussmann M., Sennefelder H., Rodin A., Maler M., Martin S.F., Goebeler M., Schmidt M. (2017). Allergy-Inducing Chromium Compounds Trigger Potent Innate Immune Stimulation Via ROS-Dependent Inflammasome Activation. J. Investig. Dermatol..

[40] Stocks S.J., McNamee R., Turner S., Carder M., Agius R.M. (2012). Has European Union legislation to reduce exposure to chromate in cement been effective in reducing the incidence of allergic contact dermatitis attributed to chromate in the UK?. Occup. Environ. Med..

[41] Bregnbak D., Johansen J.D., Jellesen M.S., Zachariae C., Menne T., Thyssen J.P. (2015). Chromium allergy and dermatitis: Prevalence and main findings. Contact Dermat..

[42] Kotlyarov A., Neininger A., Schubert C., Eckert R., Birchmeier C., Volk H.D., Gaestel M. (1999). MAPKAP kinase 2 is essential for LPS-induced TNF-alpha biosynthesis. Nat. Cell Biol..

[43] Buters J., Biedermann T. (2017). Chromium(VI) Contact Dermatitis: Getting Closer to Understanding the Underlying Mechanisms of Toxicity and Sensitization!. J. Investig. Dermatol..

[44] Gross O., Thomas C.J., Guarda G., Tschopp J. (2011). The inflammasome: An integrated view. Immunol. Rev..

[45] Watanabe H., Gehrke S., Contassot E., Roques S., Tschopp J., Friedmann P.S., French L.E., Gaide O. (2008). Danger signaling through the inflammasome acts as a master switch between tolerance and sensitization. J. Immunol..

[46] Brauer S.L., Hneihen A.S., McBride J.S., Wetterhahn K.E. (1996). Chromium(VI) Forms Thiolate Complexes with gamma-Glutamylcysteine, *N*-Acetylcysteine, Cysteine, and the Methyl Ester of *N*-Acetylcysteine. Inorg. Chem..

[47] Riche D.M., McEwen C.L., Riche K.D., Sherman J.J., Wofford M.R., Deschamp D., Griswold M. (2013). Analysis of safety from a human clinical trial with pterostilbene. J. Toxicol..

[48] Ruiz M.J., Fernandez M., Pico Y., Manes J., Asensi M., Carda C., Asensio G., Estrela J.M. (2009). Dietary administration of high doses of pterostilbene and quercetin to mice is not toxic. J. Agric. Food Chem..

[49] Kapetanovic I.M., Muzzio M., Huang Z., Thompson T.N., McCormick D.L. (2011). Pharmacokinetics, oral bioavailability, and metabolic profile of resveratrol and its dimethylether analog, pterostilbene, in rats. Cancer Chemother. Pharmacol..

[50] Sun Y., Wu X., Cai X., Song M., Zheng J., Pan C., Qiu P., Zhang L., Zhou S., Tang Z. (2016). Identification of pinostilbene as a major colonic metabolite of pterostilbene and its inhibitory effects on colon cancer cells. Mol. Nutr. Food Res..

[51] Xue E.X., Lin J.P., Zhang Y., Sheng S.R., Liu H.X., Zhou Y.L., Xu H. (2017). Pterostilbene inhibits inflammation and ROS production in chondrocytes by activating Nrf2 pathway. Oncotarget.

[52] Liu H., Zhao L., Yue L., Wang B., Li X., Guo H., Ma Y., Yao C., Gao L., Deng J. (2017). Pterostilbene attenuates early brain injury following subarachnoid hemorrhage via inhibition of the NLRP3 inflammasome and Nox2-related oxidative stress. Mol. Neurobiol..

[53] Xie Y., Xiao F., Luo L., Zhong C. (2014). Activation of autophagy protects against ROS-mediated mitochondria-dependent apoptosis in L-02 hepatocytes induced by Cr(VI). Cell Physiol. Biochem..

[54] Kosuru R., Cai Y., Kandula V., Yan D., Wang C., Zheng H., Li Y., Irwin M.G., Singh S., Xia Z. (2018). AMPK contributes to cardioprotective effects of pterostilbene against myocardial ischemia-reperfusion injury in diabetic rats by suppressing cardiac oxidative stress and apoptosis. Cell Physiol. Biochem..

[55] Hu L., Cai N., Jia H. (2017). Pterostilbene attenuates myocardial ischemia-reperfusion injury via the phosphatidylinositol 3′-kinase-protein kinase B signaling pathway. Exp. Ther. Med..

[56] Schroder M. (2008). Endoplasmic reticulum stress responses. Cell Mol. Life Sci..

[57] Verfaillie T., Rubio N., Garg A.D., Bultynck G., Rizzuto R., Decuypere J.P., Piette J., Linehan C., Gupta S., Samali A. (2012). PERK is required at the ER-mitochondrial contact sites to convey apoptosis after ROS-based ER stress. Cell Death Differ..

[58] Liu J., Fan C., Yu L., Yang Y., Jiang S., Ma Z., Hu W., Li T., Yang Z., Tian T. (2016). Pterostilbene exerts an anti-inflammatory effect via regulating endoplasmic reticulum stress in endothelial cells. Cytokine.

[59] Sirerol J.A., Feddi F., Mena S., Rodriguez M.L., Sirera P., Aupi M., Perez S., Asensi M., Ortega A., Estrela J.M. (2015). Topical treatment with pterostilbene, a natural phytoalexin, effectively protects hairless mice against UVB radiation-induced skin damage and carcinogenesis. Free Radic. Biol. Med..

[60] Sample A., He Y.Y. (2017). Autophagy in UV damage response. Photochem. Photobiol..

[61] Strozyk E., Kulms D. (2013). The role of akt/mtor pathway in stress response to UV-irradiation: Implication in skin carcinogenesis by regulation of apoptosis, autophagy and senescence. Int. J. Mol. Sci..

[62] Nagar R. (2017). Autophagy: A brief overview in perspective of dermatology. Indian J. Dermatol. Venereol. Leprol..

[63] Chen R.J., Tsai S.J., Ho C.T., Pan M.H., Ho Y.S., Wu C.H., Wang Y.J. (2012). Chemopreventive effects of pterostilbene on urethane-induced lung carcinogenesis in mice via the inhibition of EGFR-mediated pathways and the induction of apoptosis and autophagy. J. Agric. Food Chem..

