# Supplementary material for: Pterostilbene Attenuates Hexavalent Chromium-Induced Allergic Contact Dermatitis by Preventing Cell Apoptosis and Inhibiting IL-1β-Related NLRP3 Inflammasome Activation
Source: J Clin Med. 2018 Nov 27;7(12):489. doi: 10.3390/jcm7120489 (PMC6306791; doi:10.3390/jcm7120489)
Supplement: Supplementary file 1 [file jcm-07-00489-s001.pdf]

Supplementary results

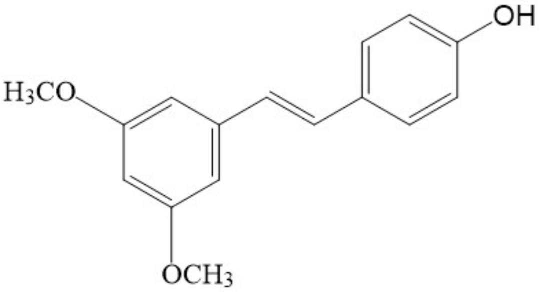

Figure S1. Chemical structure of *trans*-3,5-dimethoxy-4′-hydroxystilbene (pterostilbene).

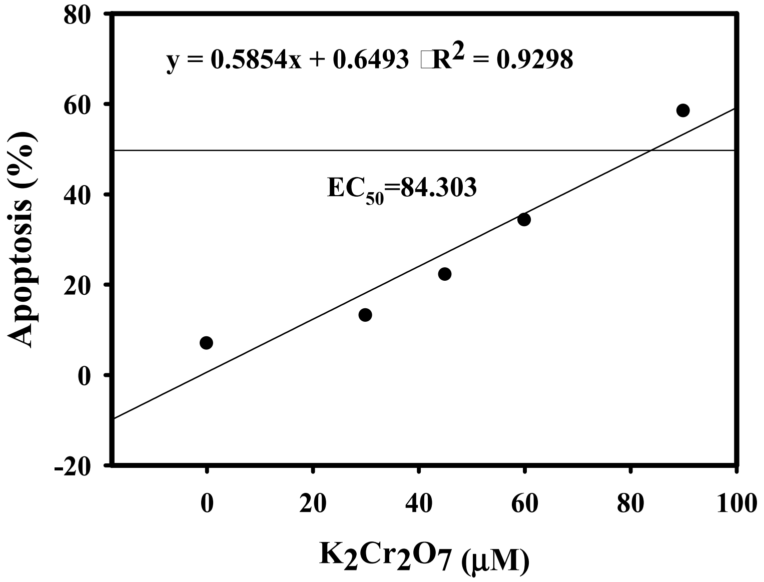

Figure S2. Percentage of apoptotic cells determined in different Cr(VI) concentrations. The EC_{50} value for apoptosis was 84.303 μM. Data presented as the mean ± standard deviation (SD) from three independent experiments.

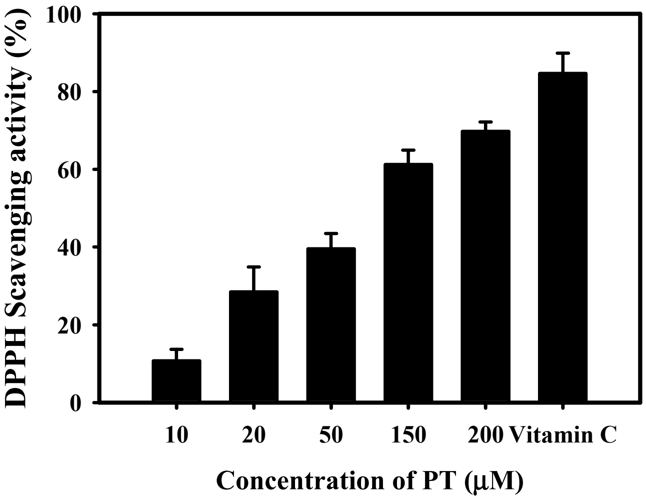

Figure S3. DPPH radical scavenging activity of PT. PT antioxidant activity was assessed via the percent inhibition (radical scavenging) of DPPH. Vitamin C (200 µM) was used as a positive control. All tests were performed in triplicate and results are expressed as means ± SD.
